# Disruption of Prostate Microvasculature by Combining Microbubble-Enhanced Ultrasound and Prothrombin

**DOI:** 10.1371/journal.pone.0162398

**Published:** 2016-09-19

**Authors:** Jinlong Zhang, Shengzheng Wu, Yongliang Liu, Lu Qiao, Wenhong Gao, Weiguo Zhang, Zheng Liu

**Affiliations:** 1 Department of Radiology, Research Institute of Surgery, Daping Hospital, Third Military Medical University, Chongqing, China; 2 Department of Ultrasound, Xinqiao Hospital, Third Military Medical University, Chongqing, China; 3 Department of Urology, Xinqiao Hospital, Third Military Medical University, Chongqing, China; 4 State key laboratory of Trauma, Burns and Combined Injury, Research Institute of Surgery, Daping Hospital, Third Military Medical University, Chongqing, China; University of Nebraska Medical Center, UNITED STATES

## Abstract

Previous studies have shown a unique method to disrupt tumor vasculature using pulsed, high-pressure amplitude therapeutic ultrasound combined with microbubbles. In this study, we attempted to destroy the prostate vasculature of canine prostates using microbubble-enhanced ultrasound (MEUS) and prothrombin. The prostates of 43 male mongrel canines were surgically exposed. Twenty-two prostates were treated using MEUS (n = 11) or MEUS and prothrombin (PMEUS, n = 11). The other 21 prostates, which were treated using microbubbles (n = 7), ultrasound (n = 7) or prothrombin (n = 7) only, served as the controls. Prothrombin was intravenously infused at 20 IU/kg. MEUS was induced using a therapeutic ultrasound device at a peak negative pressure of 4.47 MPa and a microbubble injection. Contrast-enhanced ultrasound was performed to assess the blood perfusion of the prostates. Then, the prostate tissue was harvested immediately after treatment and at 48 hours later for pathological examination. The contrast-enhanced ultrasound peak value of the prostate decreased significantly from 36.2 ± 5.6 to 27.1 ± 6.3 after treatment in the PMEUS group, but it remained unchanged in the other groups. Histological examination found severe microvascular rupture, hemorrhage and thrombosis in both MEUS- and PMEUS-treated prostates immediately after treatment, while disruption in the PMEUS group was more severe than in the MEUS group. Forty-eight hours after treatment, massive necrosis and infiltration of white blood cells occurred in the PMEUS group. This study demonstrated that PMEUS disrupted the normal microvasculature of canine prostates and induced massive necrosis. PMEUS could potentially become a new noninvasive method used for the treatment of benign prostatic hyperplasia.

## Introduction

Benign prostatic hyperplasia (BPH) is a common disease in aging men. It affects approximately 50% of men older than 50 years old and 90% of men older than 80 years old [1]. The gold standard for the treatment of BPH is transurethral resection of the prostate, which is effective but is also an invasive operation with associated surgical risks [2]. Many minimally invasive therapeutic methods, such as transurethral microwave thermotherapy, transurethral needle ablation and interstitial laser coagulation, have been developed over the past decade. However, these minimally invasive methods are inferior to transurethral resection of the prostate due to their limited relief of urodynamic obstruction [3]. Up to 62% of all patients treated by minimally invasive therapeutic methods require either medical or surgical secondary treatment within 5 years [4]. Therefore, an effective and less-invasive method for BPH treatment is desirable.

BPH develops mainly in the inner gland of the prostate [5]. Contrast-enhanced ultrasound (CEUS) has confirmed that contrast enhancement of the inner gland was much stronger than that of the outer gland [6], consistent with the elevated angiogenesis level of the inner gland [7].

Acoustic cavitation is a major physical effect of ultrasound, referring to the formation of bubbles and the oscillation of preexisting bubbles present in the propagation medium. It leads to the oscillation, expansion, compression and collapse of bubbles in the medium. When the bubbles collapse during cavitation, they can produce transient intense local heating, high pressures, microstream, shock waves, light emission, etc. Cavitation can create localized surface damage by forming fast-moving liquid jets [8, 9].

Previous studies have demonstrated that a microbubble ultrasound contrast agent can increase the possibility of acoustic cavitation and cavitation-related vascular damage effects, such as microvascular rupture and petechial hemorrhage [10–12], in which the microbubbles serve as nuclei to induce cavitation [13]. These vascular effects could be therapeutically useful in thrombolysis, disruption of the blood-brain barrier and gene or drug delivery [14–20]. At high acoustic pressure amplitudes, microbubble-enhanced ultrasound (MEUS) could cause severe mechanical damage to the endothelium of the capillaries or small vessels and could result in single vessel occlusion with the help of an intravascular injection of thrombin [21]. We also have shown that the microvasculature of a rat Walker-256 tumor could be mechanically disrupted by MEUS at a low acoustic intensity because tumor angiogenesis is a defective and vulnerable target for intravascular acoustic cavitation [22]. Therefore, it may also be possible to destroy the microvasculature of BPH using MEUS.

The general hypothesis of this study was that the microvasculature of the prostate could be disrupted using MEUS, and the vascular effects would lead to a significant reduction in prostate blood perfusion. Hemostatic injection of an agent such as prothrombin might promote the thrombotic effects. In this study, the initial successful disruption of the microvasculature of the prostate using MEUS was reported.

## Materials and Methods

### Animals

Forty-three healthy, male, mongrel dogs from 2 to 4 years old and weighing 8–14 kg were acquired from the Center for Experimental Animals of the Third Military Medical University. The sample sizes were estimated according to previous studies [23, 24]. All dogs were housed singly in stainless steel cages with a set temperature of 20–25°C and a relative humidity of about 40% to 60%. The animals were maintained on 12-h light-dark cycles. The cages were washed twice daily. Each animal was fed with the standard dog food and provided tap water ad libitum. All dogs were permitted an acclimation period of approximately 1 week.

The dogs were randomly assigned to five individual groups (Table 1) in the study. The prostates of twenty-two canines were treated using MEUS (n = 11) or MEUS and prothrombin injection (n = 11). The other 21 prostates were treated using microbubbles (MB, n = 7), ultrasound (US, n = 7) or prothrombin (n = 7) only and served as the controls.

**Table 1:** Animal group assignments and treatment protocols.

*Groups*	*TUS treatment*	*Microbubbles*	*Prothrombin*
*PMEUS (n = 11)*	*10 min*	*0*.*1 ml/kg*	*20 IU/kg*
*MEUS (n = 11)*	*10 min*	*0*.*1 ml/kg*	*0 IU/kg*
*US (n = 7)*	*10 min*	*0 ml/kg*	*0 IU/kg*
*MB (n = 7)*	*0 min*	*0*.*1 ml/kg*	*0 IU/kg*
*Prothrombin (n = 7)*	*0 min*	*0 ml/kg*	*20 IU/kg*

PMEUS, microbubble-enhanced ultrasound and prothrombin; MEUS, microbubble-enhanced ultrasound; US, ultrasound; MB, microbubbles; Prothrombin, sham ultrasound exposure and prothrombin; TUS, therapeutic ultrasound

### Ethics statement

All of the procedures were performed in accordance with the approval of the Institutional Animal Care and Use Committee of the Third Military Medical University, Daping Hospital and Xinqiao Hospital. All of the animals received care in accordance with the Guide for the Care and Use of Laboratory Animals. The animals were monitored daily throughout the experiment for heart rate, respiration rate and blood pressure. We administered an intramuscular injection of dolantin (1 mg/kg) if there were signs of pain or distress in the animals. At the end of the experiment, the animals were euthanized using an overdose of 2% sodium pentobarbital (5 ml/kg).

### Therapeutic ultrasound device

The therapeutic ultrasound (TUS) was generated by a transducer, which was comprised of an air-backed, spherically focused, 25 mm diameter concave disk (Kunshan Risheng Electronic Co., Ltd., Kunshan, China) with a 160 mm radius of curvature [22]. This transducer was driven by a wave generator and a power amplifier (Mianyang Sonic Electronic Ltd. Mianyang, China) designed for it. The transducer had a 10 mm long front chamber between the disc and the polyimide membrane, and the chamber was filled with degassed water. The geometrical focus of this transducer was exactly 150 mm from of the tip. To measure the acoustic output of the transducer at a range of 1–4 cm from the tip, a needle hydrophone (TNU001A, NTR, Seattle, WA, USA) was established, and it was adjusted by a precision 3D motion stage. This transducer was operated at the frequency of 831 KHz with a pulse length of 400 cycles and a pulse repetition frequency of 9 Hz. The acoustic pressure (peak negative pressure) output was 4.47 MPa at 10 mm from the tip. This transducer worked in an intermittent mode, with 6 seconds on and 6 seconds off. The working duty cycle was 0.22%, and the corresponding acoustic intensity (spatial peak temporal average intensity, I_SPTA_) was 0.4 W/cm^2^.

### Microbubbles

Zhifuxian [25, 26], a lipid-coated microbubble, was used for the nucleation of acoustic cavitation, as well as for the contrast agent for CEUS. It was prepared by lyophilization of two lipid suspensions, 1,2-dipalmitoyl-sn-glycero-3-phosphoglycerol (DPPG) and 1,2-distearoyl-sn–glycerol-3-phosphoethanolamine (DSPE), followed by agitation with perfluoropropane gas using a high-speed mechanical amalgamator. The size distribution and concentration of microbubbles were determined using a RC-3000 Resistance Particle Counter (OMEC Technology Co., Ltd., China). The microbubbles had a mean particle diameter of 2 μm with 98% of the particles smaller than 8 μm and a bubble concentration of 9×10^10^/mL. For CEUS, a bolus injection of 0.02 ml/kg of microbubbles was administered. For the nucleation of MEUS, a microbubble suspension of 0.1 ml/kg was constantly infused during MEUS.

### Treatment protocols

A 21-gauge needle was inserted into the veins of the upper extremities of the dogs for intravenous injection access. Anesthesia was induced by intravenous injection of 3% sodium pentobarbital at 1.0 ml/kg. After anesthesia was induced, the prostates of the animals were surgically exposed.

In the US, MB and MEUS groups, prior to treatment, some exposed prostates were imaged by two-dimensional ultrasound and CEUS using a commercial diagnostic ultrasound system (iU22, Philips ultrasound, Bothell, WA, USA) equipped with an L12-5 linear probe. The depth, gain, mechanical index and other settings were kept the same during the CEUS. In the MEUS group, eleven prostates were directly contacted and insonated for 10 min using the TUS transducer coupled with gel, while the microbubble suspension was continuously intravenously injected at 1 ml/min (0.1 ml/kg of microbubbles diluted in 10 ml of saline) at the same time. The microbubbles were replaced by 10 ml of saline in the US group. Sham TUS insonation was used in the MB group with a 10 ml microbubble injection. To prevent residual microbubbles in the circulation, TUS treatment was administered 120 min after the initial CEUS study in the US group. After treatment, CEUS was performed on the prostates again. Then, six animals in the MEUS group and four animals in the US and MB groups were euthanized with an intravenous injection of 3% sodium pentobarbital (1 ml/kg), and the prostates were harvested for acute pathological examination. The abdomens of the remaining eleven animals (including 5 from the MEUS group) were surgically closed, and the animals were kept alive for 48 hours or 4 days (MEUS, n = 2). Afterwards, those eleven prostates were collected for pathological examination.

In the PMEUS and prothrombin groups, prior to treatment, some exposed prostates were imaged by two-dimensional ultrasound and CEUS using a commercial diagnostic ultrasound system (S2000; Siemens Medical Solutions USA, Inc., Mountain View, CA, USA) equipped with a 9L4 linear array probe (frequency ranged from 4 to 9 MHz). Depth, gain and other settings were kept the same during the CEUS. Prior to TUS treatment, Human Prothrombin Complex (Shanghai RAAS Blood Products Co., Ltd) at 20 IU/kg was intravenously infused in all of the animals. In the PMEUS group, eleven prostates were directly contacted and insonated for 10 min using the TUS transducer coupled with gel, while the microbubble suspension was continuously intravenously injected at 1 ml/min (0.1 ml/kg of microbubbles diluted in 10 ml of saline) at the same time. Sham TUS insonation was used in the prothrombin group with a 10 ml saline injection. After treatment, 2 animals in the PMEUS group were injected with Evans blue (EB) for blood perfusion evaluation. In the other animals, CEUS was performed on the prostates again. Then, five animals in the PMEUS group and four animals in the prothrombin group were euthanized with an intravenous injection of 3% sodium pentobarbital, and the prostates were harvested for acute pathological examination. The abdomens of the remaining seven animals (including 4 from the PMEUS group) were surgically closed and the animals were kept alive for 48 hours. Afterwards, the seven prostates were collected for pathological examination.

### Imaging analysis

**Fig 1:**
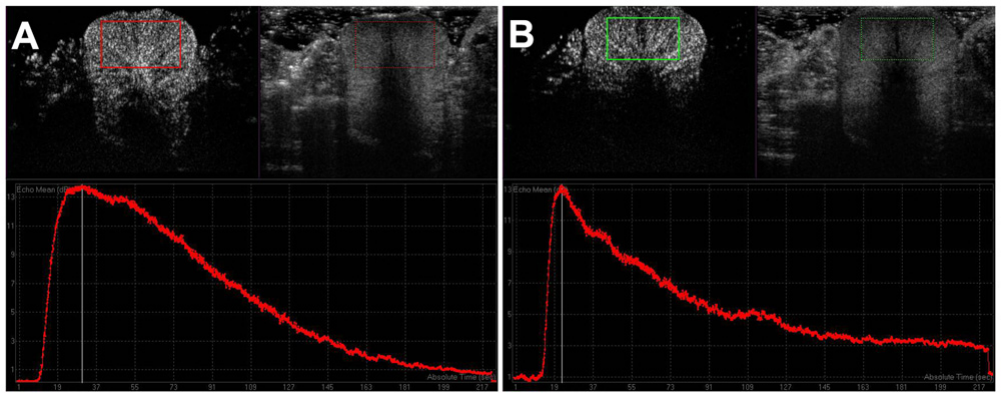
Analysis of contrast-enhanced ultrasound in the microbubble-enhanced ultrasound group. The upper panels in A (before treatment) and B (after treatment) showed the contrast-enhanced ultrasound image with two modes: contrast mode (left) and tissue mode (right). A rectangular region of interest was defined inside the frontal prostate, excluding the acoustically attenuated area. The lower panels showed a time intensity curve (TIC) generated automatically by QLAB software. (A) After a rapid ascension in acoustic intensity, the TIC showed a relative plateau around peak intensity (representing microbubble diffusion to the mesenchyme) and then a slow descent. (B) The ascension and descension of the TIC after treatment were steeper, and the diffusion plateau became imperceptible or even disappeared.

In the MB, US and MEUS groups, CEUS was analyzed for acoustic quantification using QLAB software, which was installed in iU22. A rectangular region of interest (ROI) included approximately two-thirds of the total cross-sectional area from the front of the prostate, excluding the acoustically attenuated area (Fig 1). A time-intensity curve (TIC) was generated automatically by the software. The data of peak intensity (PI) of the parenchyma, time to peak enhancement (TTP) and area under curve (AUC) were derived for acoustic quantification.

**Fig 2:**
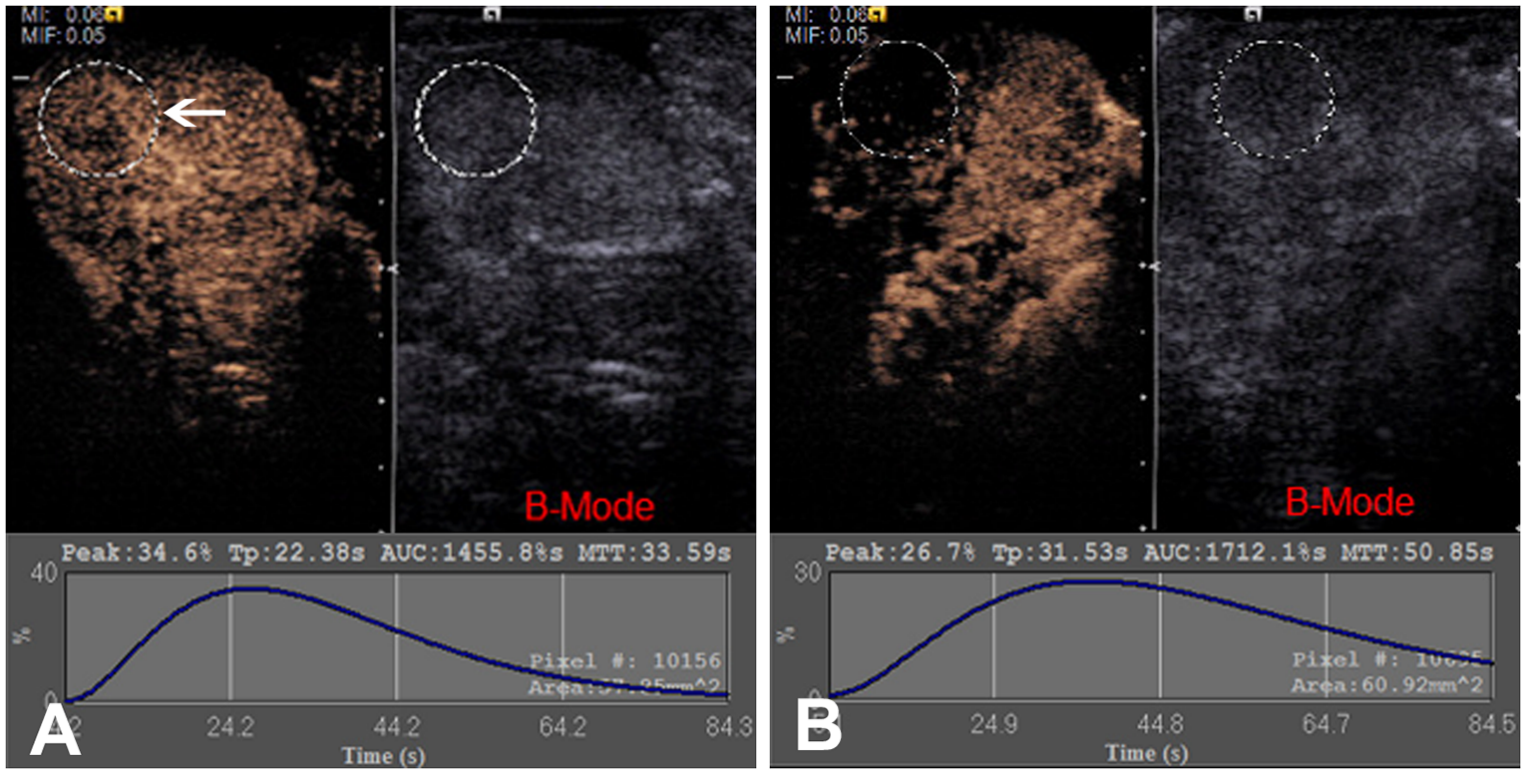
Analysis of the contrast-enhanced ultrasound in microbubble-enhanced ultrasound and prothrombin treated prostates. Interface of the acoustic quantification software showing the contrast-enhanced ultrasound and the 2-D images. (A) A spherical region of interest was set, and a time-intensity curve was analyzed before treatment. (B) In the microbubble-enhanced ultrasound and prothrombin groups, the curves after treatment show that the value of the peak decreased, compared with before treatment.

In the PMEUS and prothrombin groups, for each CEUS study, a ROI of approximately 10 × 10 mm was set as the treated region (Fig 2) using Contrast Dynamics software directly on the machine; in the treated region, a poorly enhanced area usually occurred in the PMEUS group. The location of the ROI was kept at the same location for all subsequent images. After a TIC was drawn by the software, the parameter of the peak was calculated automatically. The peak was the incremental percentage of the peak contrast intensity compared with the baseline intensity.

### Evans blue dyeing

In the PMEUS group at approximately 20 min after EB injection, 2 treated prostates were harvested. Each prostate was cut into coronal sections along the urinary track and was visualized for blood perfusion evaluation.

### Pathological examination

All of the harvested prostates were first sliced into pieces approximately 4 mm in thickness for gross examination. Then, all of the specimens obtained from the insonated prostate area were fixed in formalin, embedded in paraffin and stained with hematoxylin and eosin. The sections were observed using a light microscope.

### Statistical analysis

All of the data are expressed as values of the mean ± standard deviation. Data were subjected to Wilcoxon’s signed ranks test, which does not assume that population variances are equal. A *p* value <0.05 was deemed a significant difference. All of the data were analyzed using SPSS software, version 18.0.

## Results

All of the animals survived the processes of anesthesia, handling, CEUS and TUS treatment.

### Contrast enhanced ultrasound

**Fig 3:**
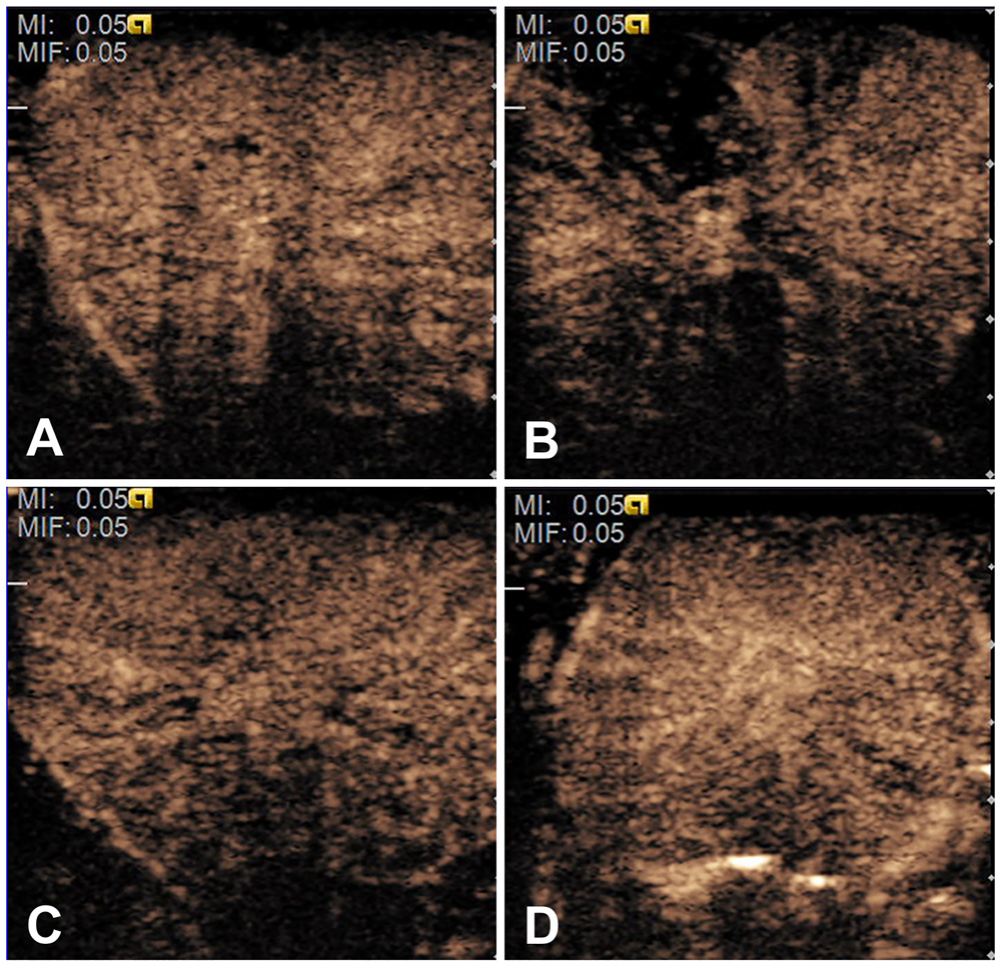
Contrast-enhanced ultrasound images of the microbubble-enhanced ultrasound and prothrombin and prothrombin-treated prostates. (B) The microbubble-enhanced ultrasound and prothrombin-treated prostate, demonstrating a significant blood perfusion decrease in the targeted area. (A, C, D) The baseline in the microbubble-enhanced ultrasound and prothrombin group (A) and all of the corresponding contrast-enhanced ultrasound images (C: before treatment, D: after treatment) of the prothrombin-treated prostates, showing homogenous blood perfusion.

Contrast enhancement of all of the prostates usually began from the peripheral major prostatic arteries and quickly spread throughout the entire gland. Before treatment, the enhancement of all of the prostate was homogenous with no non-enhanced area or perfusion defects (Fig 3A and 3C). All of the TICs obtained from digital clips before treatment demonstrated a quickly rising slope, a relative plateau of the distribution phase around the peak intensity and then a gradually descending slope (Fig 1A). In the MEUS group, the rising time shortened, and the distribution phase became imperceptible or even disappeared after treatment, with a steeper ridge of the curve (Fig 1B). The acoustic quantification of TICs resulted in the TTP being shortened by approximately 48% (*p*<0.05), while the AUC decreased by approximately 22% (*p*<0.05) after treatment (Table 2). However, no significant changes in PI were found (*p*>0.05) (Table 2). In contrast, in the PMEUS group, CEUS showed that a significantly non-enhanced or poorly perfused region was formed in the treated area immediately after treatment (Figs 2B and 3B). The ROI peak value decreased from 36.2 ± 5.6 to 27.1 ± 6.3 (Table 3). In the prothrombin group, the blood perfusion in the prostate after treatment remained homogeneous as before (Fig 3C). There were no significant differences regarding PI, TTP, AUC or the peak before or after treatment in the MB, US and prothrombin groups (Tables 2 and 3).

**Table 2:** Acoustic quantification of the time-intensity curves derived from contrast-enhanced ultrasound.

*Groups*	*TTP*		*PI*		*AUC*	
	*PRE*	*POST*	*PRE*	*POST*	*PRE*	*POST*
*MEUS*	*28*.*5±10*.*8*	*14*.*8±5*.1*	*16*.*9±1*.*8*	*17*.*1±1*.*9*	*2083*.*8±596*.*2*	*1635*.*6±524*.6*
*US*	*15*.*2±7*.*7*	*17*.*0±9*.*9*	*16*.*8±2*.*1*	*17*.*4±1*.*8*	*1933*.*8±604*.*8*	*2008*.*7±505*.*8*
*MB*	*20*.*7±3*.*1*	*20*.*9±4*.*3*	*16*.*3±2*.*3*	*16*.*1±1*.*5*	*2194*.*7±776*.*9*	*2175*.*6±688*.*1*

Data are expressed as the means ± standard deviations. MEUS, microbubble-enhanced ultrasound; US, ultrasound; MB, microbubbles; TTP, time to peak, unit in seconds; PI, peak intensity, unit in dB; AUC, means area under curve, unit in dB·s

* Indicates significant differences from the PRE values (*p*<0.05).

**Table 3:** The contrast-enhanced ultrasound peak values in the prostates before and after treatment in the microbubble-enhanced ultrasound and prothrombin group and the prothrombin group.

*Groups*	*PRE*	*POST*
*PMEUS*	*36*.*2 ± 5*.*6*	*27*.*1 ± 6*.3*
*Prothrombin*	*38*.*1 ± 7*.*0*	*37*.*9 ± 7*.*9*

Data are expressed as the means ± standard deviations. Peak, unit in %; PMEUS, microbubble-enhanced ultrasound and prothrombin; Prothrombin, sham ultrasound exposure and prothrombin

* Indicates significant differences from the PRE values (*p*<0.05)

### Evans blue dyeing

**Fig 4:**
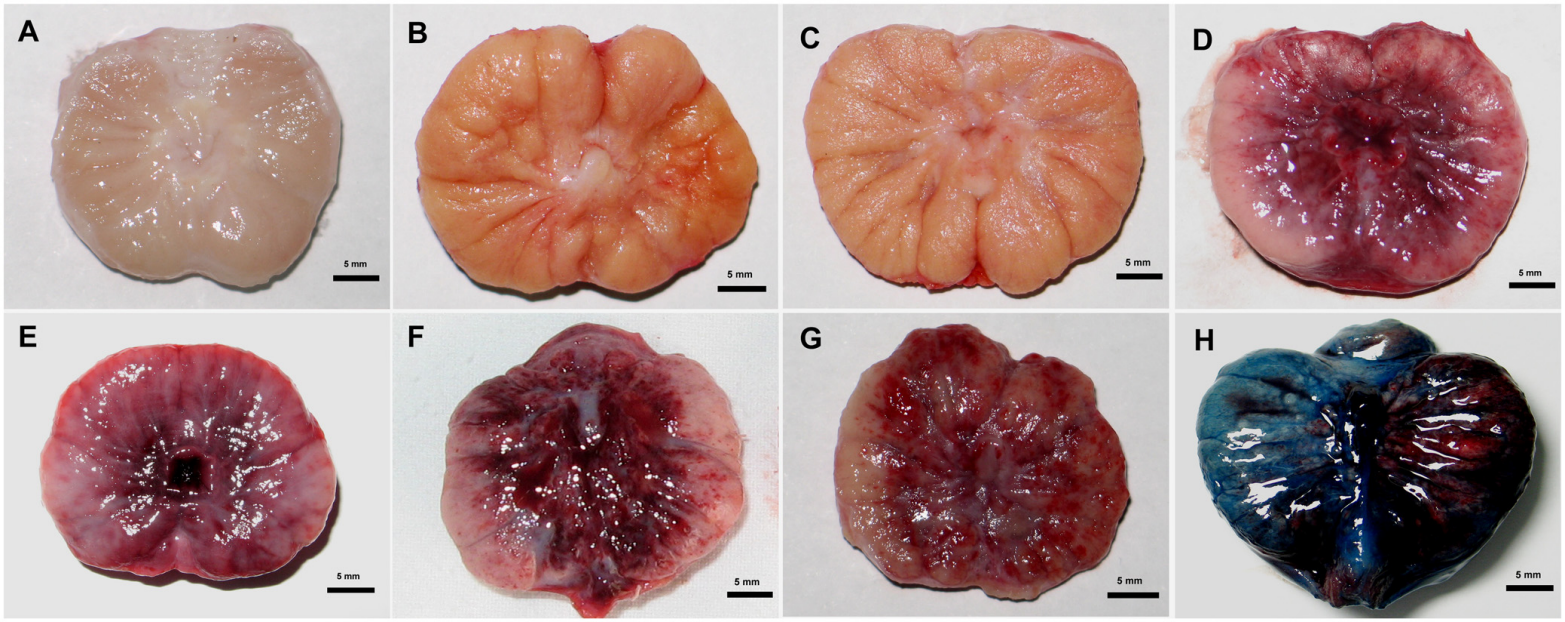
Prostate samples of the different groups obtained after treatment (scale bar = 5 mm). (A-C) Pictures from the prothrombin, microbubbles, and ultrasound-treated specimens (A: Prothrombin group, B: MB group, C: US group) demonstrated normal prostate tissue without hemorrhage. (D, E) The microbubble-enhanced ultrasound (MEUS)-treated prostate showed severe hemorrhage, especially in the tissue surrounding the urethra (D: severe hemorrhage immediately after treatment, E: hemorrhage with irregularly shaped and blurred boundaries 48 hours later). (F, G) The microbubble-enhanced ultrasound and prothrombin (PMEUS)-treated prostate showed more gross severe hemorrhage than the MEUS group throughout most of the gland, and the color was darker (F: severe hemorrhage immediately after treatment, G: diffused hemorrhage with irregular shape 48 hours later). (H) Coronal plains of the prostates with a dividing line of the urinary track demarcating the two parts after Evans blue dyeing. In the presence of microbubbles and prothrombin, the control portion (left) without ultrasound irradiation was homogeneously dyed blue. The ultrasound irradiated part (right) was dark red with severe hemorrhage, indicating that the Evans blue stain was blocked by PMEUS.

In the PMEUS group, the ultrasound irradiated part of the prostate was dark red with severe hemorrhage, and only a small amount of EB existed in the major blood vessels. In comparison, the control portion without ultrasound irradiation was homogeneously dyed blue. The dividing line of the urinary track demarcated the two parts (Fig 4H). This result showed that the blood perfusion of the prostate was partially blocked by PMEUS, consistent with the above CEUS results (Figs 2B and 3B).

### Histological examination

**Fig 5:**
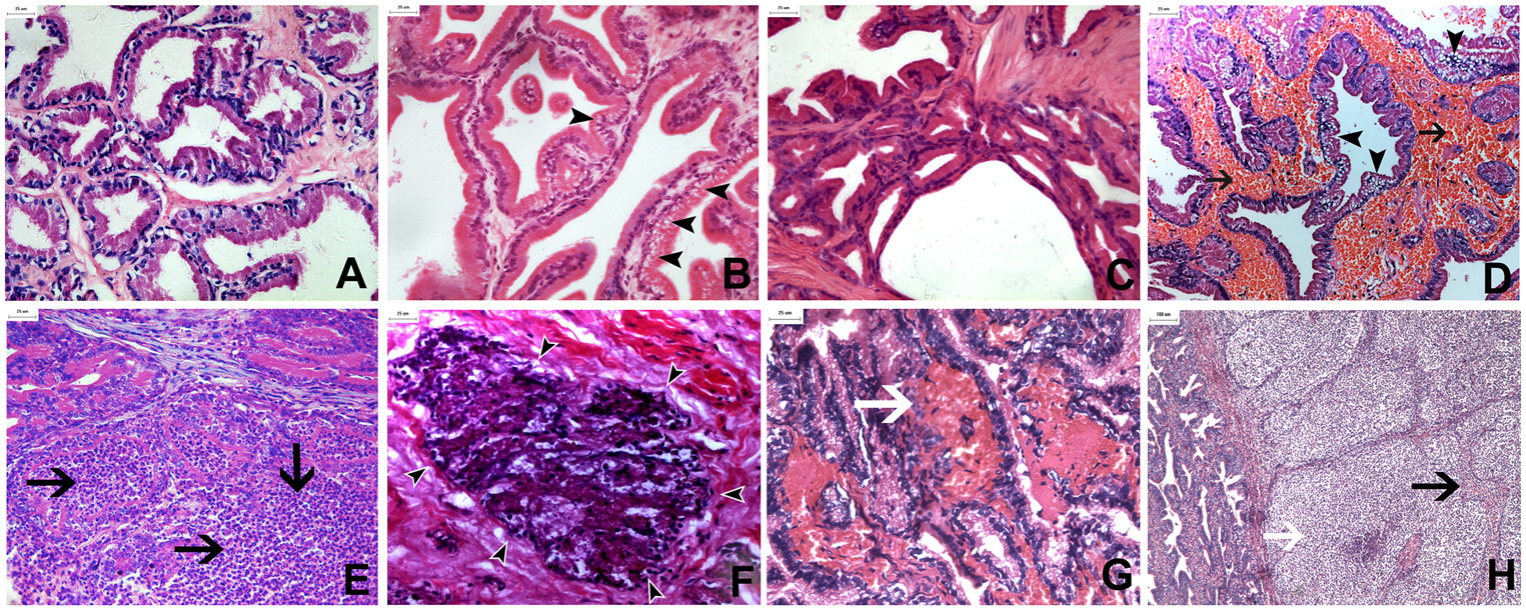
Prostate samples harvested from different groups after treatment (scale bar = 25 μm in A-G and 100 μm in H). (A) Normal histological structures displayed in the MB group. (B) Vacuolization of epithelial cells [arrowhead] was displayed in the US group immediately after ultrasound irradiation. (C) Almost complete recovery of the affected cells 48 hours later in the US group. (D-F) Microscopic examination results after microbubble-enhanced ultrasound (MEUS) treatment (D: Vacuolization of epithelial cells [arrowhead], red blood cells filling the stroma [arrow], diffuse thrombosis, enlarging and squeezing of the stroma immediately after MEUS; E: Areas of diffuse neutrophil infiltration [arrow] were observed frequently in the stroma and glandular lumen 48 hours after treatment; F: Collapse of glandular structure [arrowhead] and focal necrosis with pyknosis 4 days after treatment). (G, H) Microscopic examination results after microbubble-enhanced ultrasound and prothrombin (PMEUS) treatment (G: More severe hemorrhage and thrombosis than the MEUS group in the interstitial tissues immediately after PMEUS [white arrow]; H: Significant squeezing and focal necrosis of prostatic tissues [black arrow] by the compression of neutrophil infiltration 48 hours after treatment [white arrow].

Immediately after treatment in the MEUS group, the surface of the prostates became dark red, and the cross-sections showed severe hemorrhage (Fig 4D), especially in the surrounding urethral area. In the histological examination, severe and diffuse disruption of the microvessels led to an unidentifiable endothelium. A large number of red blood cells leaked from the vascular lumen, resulting in diffuse distribution of the microthrombi and enlarging and/or squeezing of the stroma and the glandular lumen (Fig 5D). In the PMEUS group, gross observation demonstrated diffuse and more severe hemorrhage throughout the central prostate. The hemorrhage was flaky and merged together through the prostate in the direction of the therapeutic sound beam, usually occupying more than two-thirds of the prostate section (Fig 4F). In the histological examination, we found more severe microvascular hemorrhage, hematoma and thrombosis in the interstitial tissues. Some red blood cells broke into the glandular cavities (Fig 5G).

In the prothrombin, MB and US groups, gross observation showed almost intact prostate tissue without hemorrhage immediately after treatment (Fig 4A–4C). In the histological examination, vacuolization of epithelial cells [black arrow] was found in the US group (Fig 5B). In the MB and prothrombin groups, histological examination demonstrated normal prostate tissue, in which the interstitial tissue and glandular cavities were arranged normally. There was no microvascular disruption or leakage of red blood cells in the interstitial tissue or glandular cavities or other abnormalities (Fig 5A).

Forty-eight hours after treatment, gross observation showed that the hemorrhage of the prostates became thinner and inhomogeneous in the MEUS and PMEUS groups (Fig 4E and 4G). In the histological examination, focal necrosis and diffused infiltration of neutrophils were found in the stroma and glandular lumen 48 h later in the MEUS group (Fig 5E). Furthermore, the glandular structure collapsed, accompanied by acellular substances (Fig 5F) 4 days later. In the PMEUS group, the normal glandular structure almost disappeared 48 h later in the treated area. In contrast, there was significant and diffused infiltration of inflammatory cells such as neutrophils, which filled most of the interstitium and the glandular cavities. The residual glandular epithelium and the interstitial tissues were severely compressed between the neutrophils, with patchy or flaky necrosis (Fig 5H). In the prothrombin, MB and US groups, the prostates presented as normal with no hemorrhage and with infiltration of white blood cells or necrosis 48 h later, either grossly or by histological examination (Fig 5C).

## Discussion

We demonstrated the unique method of using microbubble-enhanced acoustic cavitation to disrupt the microvasculature of the prostate. The vascular damage effects led to a significant decrease in prostate blood perfusion, resulting in aseptic inflammation and partial tissue necrosis at 48 h due to the disruption.

The current treatments for BPH are physical ablation methods, which mainly attempt to ablate or remove BPH tissues. To achieve this goal, invasive intervention is needed to deliver high energy to the prostate. These operations are invasive and sometimes risky, and it is difficult to ablate bulk tissue [2–4, 27]. None of these techniques takes advantage of the BPH vasculature as a target.

A previous study demonstrated that MEUS was capable of disrupting tumor vasculature because the defective and vulnerable tumor angiogenesis became a sensitive target under mechanic disruption by acoustic cavitation [22]. We hypothesized that MEUS could reproduce the vascular disruption effects in BPH. The BPH vasculature consists of highly angiogenetic vascularization [7] similar to tumor angiogenesis. The high microvessel density corresponds to the highly contrasting perfused inner gland in CEUS [6], which could become a sensitive therapeutic target for MEUS because the high density of the microbubble contrast agent could provide sufficient cavitation nuclei for MEUS destruction.

In this experiment, MEUS was induced using a therapeutic ultrasound device, which had previously been used in tumor vessel destruction. It is characterized by high acoustic pressure amplitude with pulsed and intermittent ultrasound bursts. Because the device works with a low duty cycle and at a low intensity level, the thermal effects of ultrasound are minimal and negligible. Therefore, the mechanical effects (such as micro-jets, shock waves, etc.) released from acoustic cavitation are considered to be the only possible mechanism of treatment.

The abnormal profile of the TICs after MEUS represented a lack of diffusion time, which might indicate destroyed microcirculation, where contrast agents were distributed from major arteries to capillaries. PI, representing the blood perfusion, showed no significant changes, compared with before treatment. The shortened TTP showed that the blood inflow circulating in the major arteries was accelerated, which caused a significant decrease in the AUC after MEUS. These effects were expected to be the results of physical stimulation by cavitational activity.

For the first time, we discovered that the microvasculature of the prostate could be substantially disrupted by MEUS. Severe microvascular rupture, hemorrhage, hematoma and thrombosis were observed in the prostate tissues as the results of disruption. Prothrombin was added to induce more thrombosis. Hematoma and thrombosis were distributed mainly within the interstitial tissues, and they sometimes broke into the glandular cavities due to vascular rupture and glandular epithelial damage (Fig 5D and 5G). These histological changes could form joint compression that reduced the blood perfusion of the prostate. EB is an azo dye with a high affinity for serum albumin, and it can be applied to assess blood perfusion. The blood perfusion reduction could be confirmed by the results of CEUS and EB dyeing, which were the visually poorly enhanced targeted area, the significant decrease in the peak value and the poor EB dyeing in the PMEUS-treated prostates. In this experiment, we did not acquire a complete shutdown of prostatic blood perfusion like we did with tumor treatment [22]. Tumor angiogenesis develops rapidly with many intrinsic defects [28], and it proved to be a sensitive and vulnerable target for MEUS attack. Human BPH also overexpresses vascular growth factors and grows with similar angiogenesis [7]. However, the prostates in this experiment appeared to be normal without BPH. Normal vessels evolve with an integrated and robust wall. MEUS-induced vascular effects on normal vessels were limited to endothelial malformation or microvascular wall rupture and not vasculature destruction or blood flow obstruction [21, 29]. Nevertheless, the effects did induce a flow reduction and flaky necrosis of the prostates. We expect that MEUS could produce more severe vascular disruption in human BPH and prostate cancer because their vasculatures are angiogenetic.

In our experiment, we did not obtain a significant flow reduction in only the MEUS-treated prostates, as we expected. Therefore, prothrombin was added to the formal experiment to induce more thrombosis and flow reduction when the vascular damage occurred. It was obvious that no such effects occurred in the three control groups. For US treatment, there was not a sufficient amount of microbubbles to nucleate the acoustic cavitation. The cavitation activity was expected to be weak without injected microbubbles. Prothrombin injection without MEUS could not find the vascular injury to cause its activation.

Prothrombin is a combination of several blood clotting factors, including factors II, VII, IX and X. It facilitated intravascular thrombosis by activating more coagulation in this study. However, there are risks associated with high dose prothrombin use, such as pulmonary embolism, myocardial and renal infarction, limb ischemia and deep vein thrombosis [30]. Therefore, prothrombin at a dose of 20 IU/kg, which is clinically approved for intravenous injection, was used in our study.

This combined PMEUS treatment is different from histotripsy. Histotripsy uses a large focusing device and a much higher acoustic pressure of 4.5 to 22 MPa [31, 32]. Our device is small and portable, and it operates with much less acoustic intensity. Histotripsy destroys the entire target tissue, while MEUS disrupts only the vasculature.

PMEUS is also distinguished from high intensity focused ultrasound and other thermal ablation techniques. These techniques ablate BPH using high local heat deposits, usually greater than 60°C [2, 33, 34]. However, our device could not generate a temperature increase because of the low duty cycle and the low acoustic intensity. Therefore, we believe that cavitation was the only mechanism of vascular damage. This technique exploits the prostate neovasculature as a target, overcoming the side effects of thermal ablative techniques.

Due to the flow reduction and vascular trauma, PMEUS led to prostatic ischemia and aseptic inflammation, resulting in patchy necrosis and the infiltration of neutrophils. We presume that BPH tissues could be partially ablated, relieving obstruction of the urinary tract, along with the absorption of necrotic tissues or inflammation.

Despite the findings, this preliminary study had many limitations. For example, two different types of ultrasound equipment were used to evaluate blood perfusion of the prostate in this experiment. Additionally, we did not evaluate the long-term histological effects of PMEUS treatment on the prostate, which are future steps of this study.

## Conclusions

In summary, we found that MEUS could disrupt the microvasculature of the prostate, resulting in massive prostatic necrosis. This simple and presumably noninvasive therapeutic method could become a new physical therapy for BPH.
